# Supplementary material for: Provision of Microbiology, Infection Services and Antimicrobial Stewardship in Intensive Care: A Survey across the Critical Care Networks in England and Wales
Source: Antibiotics (Basel). 2023 Apr 17;12(4):768. doi: 10.3390/antibiotics12040768 (PMC10135214; doi:10.3390/antibiotics12040768)

University Hospital Southampton
NHS Foundation Trust

Antibiotic use in ICU- A Survey of intensive care physicians

* 1. Your Hospital Name

* 2. What is your grade?

Other (please specify)

* 3. Is your ICU a specialist or general unit?

Type of Unit

Specify if other specialist unit

* 4. How many standard ICU beds do you have?

5. Do you use antibiotic guidance in ICU?

- Local specific ICU guidance?
- Hospital/ Trust based guidance?
- National guidance?
- No guidance?
- Other (please specify)

6. Do you have access to the local bacterial epidemiology data? e.g., susceptibility profiles, multi-drug resistant bacteria rates – MRSA, ESBL, CPE)

- ◯ Yes
- ◯ No
- ◯ Not sure

If yes, please comment on your strategy (E.g. How this surveillance data is acquired and how do you access this data?)

7. Is the antibiotic prescription fully electronic in your service?

- ◯ Yes
- ◯ No
- ◯ Unsure
- ◯ Other (please specify)

8. Do you have a dedicated infection control prevention nurse in ICU?

- ◯ Yes
- ◯ No
- ◯ Don't Know
- ◯ Other (please specify)

9. Do you have a dedicated microbiologist/ infectious diseases specialist who provides regular input in your ICU?

- ◯ Yes
- ◯ No
- ◯ Other (please specify)

10. How often does your microbiologist/infectious diseases specialist do a ward round?

- ◯ Daily
- ◯ Alternate days
- ◯ Three times weekly
- ◯ No regular ward rounds, only telephone advice
- ◯ No regular ward rounds and no telephone advice
- ◯ No microbiology or infectious diseases service
- ◯ Other (please specify)

11. When you prescribe empirical antibiotics, are they duration limited?

- ◯ Yes, for all antibiotics
- ◯ Yes, for some antibiotics
- ◯ No

Other (please specify)

12. What is your local practice of standard duration of antibiotics for the following conditions?

	1-3 days	4-5 days	6-7 days	8-10 days	11-14 days
Septic shock	◯	◯	◯	◯	◯
Community acquired pneumonia	◯	◯	◯	◯	◯
Ventilator associated pneumonia	◯	◯	◯	◯	◯
Hospital acquired penumonia	◯	◯	◯	◯	◯
Proven line infection (post line removal)	◯	◯	◯	◯	◯
Primary intra-abdominal sepsis (source controlled)	◯	◯	◯	◯	◯
Community acquired urinary sepsis (lower UTI)	◯	◯	◯	◯	◯
Community acquired urinary sepsis (upper UTI)	◯	◯	◯	◯	◯

13. Do you have routine access to any of the following?

- ☐ C- reactive protein (CRP)
- ☐ Procalcitonin (PCT)
- ☐ Pro-Adrenomodullin (ProADM)
- ☐ Beta-D-glucan
- ☐ IL6
- ☐ TNF Alpha
- ☐ Rapid molecular PCR platform for single/multiple respiratory pathogen (results within 2 hours)
- ☐ Rapid molecular PCR platform for single/multiple respiratory pathogen (results <24 hrs)
- ☐ Other (please specify)

14. Do you have access to local antibiotic consumption data? If so, do you discuss this data routinely in a multi-disciplinary meeting?

- ◯ Data available and discussed in a multi-disciplinary meeting
- ◯ Data available and NOT discussed in a multi-disciplinary meeting
- ◯ Data not available
- ◯ Unsure
- ◯ Other (please specify)

15. What data management system do you have to audit patients who have had antibiotics?

- ☐ Case based data collection- electronic
- ☐ Case based data collection- manual
- ☐ None
- ☐ Not sure

Other (please specify)

16. Do you participate in ICU clinical research?

- ◯ Yes
- ◯ No
- ◯ I Don't know
- ◯ Other (please specify)

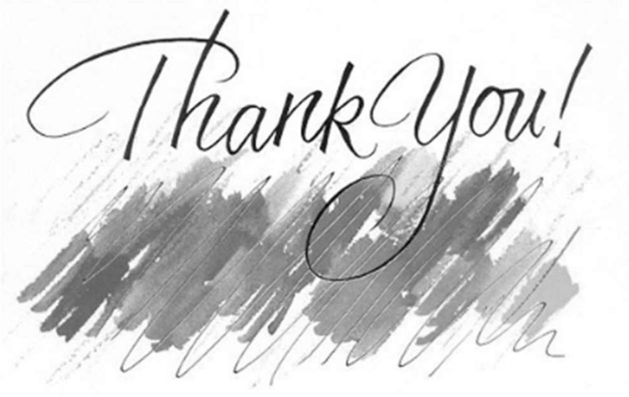

Supplement: Supplementary file 1 [file antibiotics-12-00768-s001.zip › antibiotics-2301080-supplementary.pdf]
